# Leveraging generative modeling to analyze multiple related cryo-EM datasets

**DOI:** 10.1063/4.0000874

**Published:** 2025-10-27

**Authors:** Maria V Carreira, Laurel F Kinman, Joseph H Davis

**Affiliations:** 1 MIT, Cambridge, MA, USA; 2 UCSF, San Francisco, CA, USA

## Abstract

Cellular proteomes are diverse and dynamic. To carry out their biological function and adapt to changing environments, protein and higher-order complexes must alter their composition and conformation, resulting in structural heterogeneity. Studying such structural heterogeneity of protein machines is thus of great interest, and the emergent application of generative modelling tools in single-particle cryo-electron microscopy (cryo- EM) has proved to be powerful not only to determine near-atomic resolution structures of biological molecules, but also to decipher their conformational and compositional landscape, shedding light on complex biological processes. Typically, related cryo-EM datasets are processed in isolation, and the structural impact of perturbations (e.g., mutations, treatment conditions, time-series, ligands, etc.) are inferred from the resulting isolated structures. Although this approach can be useful, the joint analysis of related samples has the potential to improve both the resolution of the resulting structures, and aid in interpreting the impact of the perturbations, leveraging the information content shared by multiple related datasets. Considering this, there is a need for more general tools and approaches to jointly analyze related datasets and directly incorporate information of the perturbation into the analysis process. Here, we present multiDRGN, an augmented cryoDRGN framework, to integrate heterogeneous reconstructions of multiple related cryo-EM datasets. Notably, multiDRGN includes an additional conditional-encoding variable in the form of per-dataset labels. We test the efficacy of imposing priors that consider the sample condition in jointly analyzing related datasets and assess model performance using a combination of synthetic ‘ground truth’ and real datasets.

